# Structure–function characterization of two enzymes from novel subfamilies of manganese peroxidases secreted by the lignocellulose-degrading Agaricales fungi *Agrocybe pediades* and *Cyathus striatus*

**DOI:** 10.1186/s13068-024-02517-1

**Published:** 2024-06-01

**Authors:** María Isabel Sánchez-Ruiz, Elena Santillana, Dolores Linde, Antonio Romero, Angel T. Martínez, Francisco Javier Ruiz-Dueñas

**Affiliations:** Centro de Investigaciones Biológicas Margarita Salas (CIB), CSIC, Ramiro de Maeztu 9, 28040 Madrid, Spain

**Keywords:** *Agrocybe pediades*, *Cyathus striatus*, Agaricales fungi, Lignin, Manganese peroxidase, Novel enzymatic subfamilies, Enzyme catalysis, Crystal structure, Manganese oxidation site

## Abstract

**Background:**

Manganese peroxidases (MnPs) are, together with lignin peroxidases and versatile peroxidases, key elements of the enzymatic machineries secreted by white-rot fungi to degrade lignin, thus providing access to cellulose and hemicellulose in plant cell walls. A recent genomic analysis of 52 Agaricomycetes species revealed the existence of novel MnP subfamilies differing in the amino-acid residues that constitute the manganese oxidation site. Following this in silico analysis, a comprehensive structure–function study is needed to understand how these enzymes work and contribute to transform the lignin macromolecule.

**Results:**

Two MnPs belonging to the subfamilies recently classified as MnP-DGD and MnP-ESD—referred to as Ape-MnP1 and Cst-MnP1, respectively—were identified as the primary peroxidases secreted by the Agaricales species *Agrocybe pediades* and *Cyathus striatus* when growing on lignocellulosic substrates. Following heterologous expression and in vitro activation, their biochemical characterization confirmed that these enzymes are active MnPs. However, crystal structure and mutagenesis studies revealed manganese coordination spheres different from those expected after their initial classification. Specifically, a glutamine residue (Gln333) in the C-terminal tail of Ape-MnP1 was found to be involved in manganese binding, along with Asp35 and Asp177, while Cst-MnP1 counts only two amino acids (Glu36 and Asp176), instead of three, to function as a MnP. These findings led to the renaming of these subfamilies as MnP-DDQ and MnP-ED and to re-evaluate their evolutionary origin. Both enzymes were also able to directly oxidize lignin-derived phenolic compounds, as seen for other short MnPs. Importantly, size-exclusion chromatography analyses showed that both enzymes cause changes in polymeric lignin in the presence of manganese, suggesting their relevance in lignocellulose transformation.

**Conclusions:**

Understanding the mechanisms used by basidiomycetes to degrade lignin is of particular relevance to comprehend carbon cycle in nature and to design biotechnological tools for the industrial use of plant biomass. Here, we provide the first structure–function characterization of two novel MnP subfamilies present in Agaricales mushrooms, elucidating the main residues involved in catalysis and demonstrating their ability to modify the lignin macromolecule.

**Supplementary Information:**

The online version contains supplementary material available at 10.1186/s13068-024-02517-1.

## Background

Plant biomass constitutes the most abundant organic carbon source in land ecosystems [1]. Lignocellulose biorefineries seek the integral utilization of this biomass as a renewable feedstock to produce chemicals, fuels and materials in more sustainable and environmentally friendly industrial processes compared to classical petroleum-based refineries [2]. This involves an efficient use of the plant cell-wall polymers, cellulose, hemicellulose and lignin from wood, straw, grasses, etc. Today, the use of lignocellulosic feedstock is mostly focused on cellulose and hemicellulose fractions for which lignin must be removed. However, conversion of the latter fraction of plant biomass into valuable products is crucial for the economic viability of lignocellulose biorefineries [3, 4]. Indeed, lignin is the largest natural source of aromatic carbon and can be an invaluable renewable source of aromatic molecules to be used as platform chemicals or active ingredients [5, 6]. In both cases—whether to degrade lignin and give access to cellulose and hemicellulose or to depolymerize it into simpler aromatic compounds—new biocatalytic tools (i.e., microorganisms and enzymes) need to be identified, and new processes developed, to meet the objectives of current policies aimed at fostering a sustainable biobased economy [7].

Lignin is synthesized by the oxidative radical polymerization of three major *p-*hydroxycinnamyl alcohols (so-called monolignols), which are at the origin of its *p-*hydroxyphenyl, guaiacyl and syringyl units [8], together with other more recently discovered monomers [9]. These units are linked to each other by different ether and carbon–carbon bonds forming the complex structure of the lignin macromolecule. Moreover, lignin composition varies among different groups of vascular plants. Thus, woody gymnosperms (softwoods) have the highest lignin content, and their lignin is made up mostly of guaiacyl units. In contrast, lignin of woody angiosperms (hardwoods) consists of syringyl and guaiacyl units, while that from non-woody angiosperms also contains *p-*hydroxyphenyl units. All these types of lignin protect cellulose and hemicellulose from degradation and are difficult to break down due to their aromatic nature and heterogeneous structure [10].

A specific group of Agaricomycetes species, known as white-rot fungi, has developed mechanisms that allow them to overcome the recalcitrance of the lignin polymer [11]. Their efficient depolymerization of lignin plays a major role in Earth’s carbon cycle [11, 12], and makes these fungi immensely valuable for biotechnological applications targeting the industrial utilization of plant biomass [13, 14]. The way white-rot fungi degrade this complex polymer has been described as an “enzymatic combustion” [15], in which peroxidases of class-II (PODs) of the heme peroxidase-catalase superfamily [16] (Family AA2 in CAZY database, http://www.cazy.org) lead this intricate process, which also involves phenol-oxidizing laccases (Family AA1 in CAZY database) and other oxidoreductases [12]. These peroxidases are classified as manganese peroxidases (MnPs), lignin peroxidases (LiPs) and versatile peroxidases (VPs), and differ mainly in their catalytic sites [17].

MnPs are the evolutionary origin of the other ligninolytic peroxidase families [18]. They are the most common lignin-modifying peroxidases produced by ligninolytic fungi. Several mechanisms have been proposed by which MnPs can initiate delignification of plant biomass [19]. The primary mechanism involves the MnP oxidation of Mn^2+^ to Mn^3+^ and the subsequent formation of chelates with fungal dicarboxylic acids. These chelates act as diffusible redox-mediators that can oxidize the phenolic units of lignin [20]. Another described mechanism of indirect lignin degradation by MnPs is based on their ability to promote lipid peroxidation [21]. Thus, in the presence of Mn^2+^ and unsaturated fatty acids, MnPs can form lipid peroxy radicals that diffuse and oxidize the major non-phenolic fraction of lignin. Finally, certain types of the short MnPs mentioned below could directly oxidize phenolic compounds (in the absence of mediators) [22], as previously shown for a mutated VP engineered into a short MnP [23], providing to these MnPs the ability to oxidize lignin phenolic units in direct contact with the heme cofactor.

Regarding their structure, MnPs can be classified based on the length of their C-terminal tail, which has a direct effect on the catalytic properties and stability of these enzymes [24]. Thus, members of the long MnP subfamily present a C-terminal tail that contributes to the oxidation of Mn^2+^ at the internal heme propionate and prevents direct oxidation of bulky substrates, while improving their stability at acidic pH. These MnPs efficiently oxidise Mn^2+^ thanks to their high affinity for this cation [24, 25]. In contrast, short MnPs lack this tail, less efficiently oxidize Mn^2+^ [22], and their internal heme propionate is more exposed to the solvent. This allows these enzymes to catalyze direct oxidation of compounds other than manganese ion at this site as mentioned above [24].

MnP enzymes could also be classified in different subfamilies based on the composition of the manganese coordination sphere. Thus, the initially characterized short/long MnPs [26] contain three acidic residues (two glutamates and one aspartate) close to the internal heme propionate that can bind Mn^2+^ [27]. More recently, in a genomic analysis of 52 Agaricomycetes species, new subfamilies of MnPs have been identified in Agaricales and some Russulales fungi, with “atypical” residues forming their putative manganese binding sites [28]. These novel subfamilies would include (with putative Mn^2+^-binding residues in parentheses) MnP-ESD (glutamic/serine/aspartic), MnP-DGD (aspartic/glycine/aspartic) and MnP-DED (aspartic/glutamic/aspartic). Although some of these enzymes have been occasionally identified (mainly in genomes) [29–31], there is still a lack of information about their structural features, and catalytic properties.

In the present study, two MnPs belonging to two of the aforementioned novel MnP subfamilies were found to be actively secreted by the Agaricales fungi *Agrocybe pediades* and *Cyathus striatus* while growing on lignocellulosic materials. Heterologous expression and in vitro activation enabled the subsequent structural–functional characterization of both enzymes, being the first time that enzymes from these subfamilies have been crystallyzed.

## Results

### Peroxidases produced by *A. pediades* and *C. striatus* in lignocellulolytic cultures

*A. pediades* and *C. striatus* were grown on wheat (*Triticum aestivum*) straw and beech (*Fagus sylvatica*) wood, respectively (lignocellulolytic conditions). The extracellular proteins secreted by both fungi were collected after 14 days of incubation and analyzed by nano-liquid chromatography–tandem mass spectrometry of the total peptides resulting from trypsin hydrolysis. The diversity and relative abundance of ligninolytic PODs identified in both secretomes were compared to each other and to those found in control cultures grown in glucose–ammonium medium (Table 1). For that, a semi-quantitative analysis was performed based on the peptide-spectrum match values of each of the peroxidases identified by at least two unique peptides. *A. pediades* PODs were identified in the secretome of wheat-straw cultures: one each from the novel MnP-DGD and MnP-ESD subfamilies [28] and one LiP, with only one MnP (JGI-ID#715400) being selectively produced on this lignocellulosic cultures. By contrast, *C. striatus* secreted PODs only when grown on beech wood, all belonging to the MnP-ESD subfamily. Among the peroxidases produced by these fungi, we selected for further study two representatives of two MnP subfamilies not yet characterized: i) JGI-ID#715400 from *A. pediades* (hereinafter referred to as Ape-MnP1); and ii) JGI-ID#1413095 from *C. striatus* (hereinafter referred to as Cst-MnP1). These enzymes were the main PODs secreted in lignocellulose cultures after 14 days of incubation.

**Table 1:**
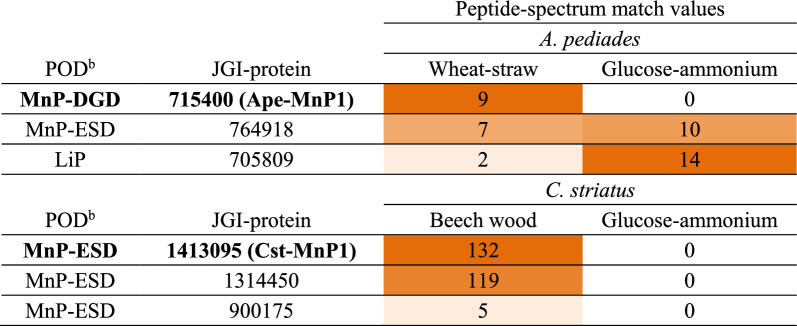
POD diversity and abundance in the secretomes of *A. pediades* and *C. striatus*^a^

^a^Enzymes were sorted from highest to lowest abundance based on total peptide-spectrum match values in 14-day cultures on lignocellulose (wheat straw or beech wood) or in glucose–ammonium medium*.* Color goes from dark orange for the enzyme with the highest abundance to white when an enzyme is absent. The names of enzymes selected for further characterization are highlighted in bold

^b^MnP subfamilies named according to Ruiz-Dueñas et al. [28] based on the amino-acid residues putatively forming their manganese oxidation sites (Asp, Gly and Asp in MnP-DGD; and Glu, Ser and Asp in MnP-ESD). Note that this nomenclature is changed later in the light of the results obtained in the present study (with MnP-DDQ and MnP-ED replacing MnP-DGD and MnP-ESD, respectively)

### Heterologous expression, enzyme activation and purification of two novel MnPs

Ape-MnP1 and Cst-MnP1 were heterologously expressed as inclusion bodies in *Escherichia coli*. A total of 672 folding conditions were explored for optimization of their in vitro activation, in which different urea, oxidized glutathione (GSSG), hemin and glycerol concentrations, as well as different temperature and pH values were tested using constant concentrations of dithiothreitol [DTT] (0.1 mM), ethylenediaminetetraacetic acid [EDTA] (20 µM), CaCl_2_ (5 mM) and protein (0.1 mg/mL). After larger scale activation under the conditions optimized for Ape-MnP1 (i.e. 0.16 M urea, 0.8 mM GSSG, 15 μM hemin, 50 mM Tris–HCl, pH 9, and 24 h incubation at 4 °C) and Cst-MnP1 (i.e. 0.6 M urea, 0.8 mM GSSG, 20% glycerol, 5 μM hemin, 50 mM Tris–HCl, pH 9.5, and 72 h incubation at 4 °C) (shown in Additional file 1: Fig. S1A, B), the enzymes were purified in a single anion-exchange chromatographic step (Additional file 1: Fig. S1C, D). Both peroxidases showed a molecular mass in agreement with their theoretical values (~ 37.8 kDa for Ape-MnP1 and ~ 36.8 kDa for Cst-MnP1) and a Reinheitzahl value (*A*_410_/*A*_280_) of ~ 5.7 which was used as a criterion of purity. Their UV–visible spectra (Additional file 1: Fig. S2) were characteristic of properly folded peroxidases with the heme cofactor in the active site. The Soret bands with maxima at 408 nm and charge transfer bands (CT1 at 637 nm and CT2 at 502 nm) corresponded to resting-state enzymes containing a high-spin ferric heme [22, 32, 33].

### Crystal structures of two enzymes representative of two novel MnP subfamilies

The molecular structures of Ape-MnP1 and Cst-MnP1 crystallized in the presence of Mn^2+^ were successfully solved at 1.50 and 1.24 Å resolution, respectively (Protein Data Bank, PDB entries 8qx0 and 8qwt). The crystallographic structure of Ape-MnP1 in the absence of this cation was also determined at 1.60 Å resolution (PDB entry 8qwx). Crystallographic data collection and refinement statistics are provided in Table 2. The refined structures of the two MnPs with manganese yielded near complete models. Thus, the model of Ape-MnP1 consists of 335 amino acids (from Thr1 of the mature protein to Asp335) and that of Cst-MnP1 includes 331 residues (from Val1 of the mature protein to Ser331). Only the last two residues of the C-terminal end were missing in Ape-MnP1 (Asp336 and Ser337). Unlike these, the structure of Ape-MnP1 in the absence of manganese lacked the last five C-terminal residues (Gln333–Ser337). This suggests that the amino acids located in this region exhibit certain mobility that could be restricted by the presence of manganese.

**Table 2:** X-ray crystallographic data and refinement statistics

	Cst-MnP1	Ape-MnP1	Mn-free Ape-MnP1
Wavelength (Å)	0.9792	0.9792	0.9792
Space group	P212121	P21	P21
*Unit cell dimensions*			
*a*	47.79 Å	62.63 Å	62.62 Å
*b*	56.64 Å	39.81 Å	39.63 Å
*c*	109.51 Å	63.56 Å	63.49 Å
*α*	90°	90°	90°
*β*	90°	101.5°	101.23°
*γ*	90°	90°	90°
Resolution (Å)	56.6–1.5	62.3–1.24	48.7–1.6
*R*_meas_^a^	0.136 (1.37)	0.068 (0.70)	0.061 (0.57)
*R*_pim_	0.076 (0.77)	0.037 (0.38)	0.041 (0.43)
*I*/*σI*	8.3 (1.8)	10.0 (1.7)	14.1 (2.4)
Completeness (%)	99.9 (99.8)	95.2 (94.9)	98.3 (98.3)
Redundancy	5.8 (5.8)	3.2 (3.2)	3.3 (3.3)
CC_1/2_^b^ (%)	99.6 (63.8)	99.9 (54.0)	99.8 (80.1)
No. unique reflections	48,414 (2337)	81,601 (3964)	39,894 (1967)
*R*_work_/*R*_free_	0.16/0.19	0.18/0.20	0.15/0.19
No. atoms	2692	2712	2739
Protein	2419	2459	2447
Ligand	102	73	85
Water	171	180	207
B-factors (Å^2^)	18.52	13.73	18.6
Protein	17.54	13.18	17.65
Ligand	30.71	17.76	29.20
Water	25.06	19.61	25.47
	*R.m.s deviations*		
Bond lengths (Å)	0.010	0.013	0.011
Bond angles (°)	1.84	1.95	1.87
	*Ramachandran*		
Favored (%)	95.74	98.80	98.79
Allowed (%)	3.65	1.20	1.21
Outliers (%)	0.61	0.00	0.00
PDB codes	8QWT	8QX0	8QWX

Statistics for the highest-resolution shell are shown in parenthesis

^a^*R*_meas_ = Σ_*hkl*_ (*n*/*n* − 1)^1/2^Σ_*i*_ |*I*_*i*_(*hkl*) − <|> (*hkl*)/Σ_*hkl*_Σ_*i*_*I*_*i*_(*hkl*), where *I*_*i*_(*hkl*) is the intensity measured for the *i*th reflection and < *I* > (*hkl*)) is the average intensity of all reflections with indices *hkl*

^b^CC_1/2_ is the correlation coefficient between two random half datasets

**Fig. 1:**
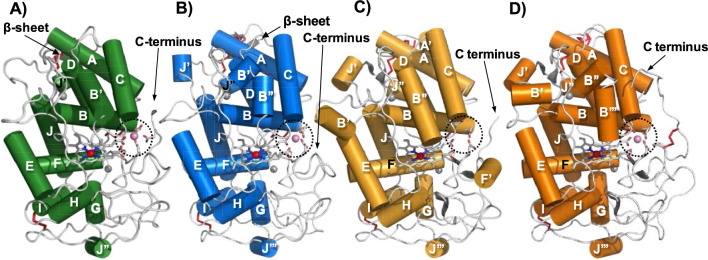
Overal crystal structures of Ape-MnP1 (**A**), Cst-MnP1 (**B**), Pos-MnP4 (**C**), and Pch-MnP1 (**D**). The schemes show α-helices (cylinders), antiparallel β-strands (grey), the heme cofactor (CPK sticks) with the iron center (red sphere), two structural calcium ions (grey spheres), disulphide bonds (red sticks), and one Mn^2+^ ion (pink sphere in dotted circle) in those structures crystallized in the presence of this metal (PDB entries 8qx0, *A. pediades* Ape-MnP1; 8qwt, *C. striatus* Cst-MnP1; 4bm1, *Pleurotus ostreatus* Pos-MnP4; and 3m5q, *Phanerochaete chrysosporium* Pch-MnP1, respectively)

Besides the amino acids of the polypeptide sequences, final models, crystallized in the presence of manganese, contain a heme prosthetic group, two structural calcium ions, and a substrate Mn^2+^ ion (Fig. 1a, b), as well as 180 (Ape-MnP1) and 171 (Cst-MnP1) solvent molecules (with three Mg^2+^ ions from the crystallization medium also present in the Cst-MnP1 structure). The substrate-free Ape-MnP1 model differs from the substrate-bound enzyme in the number of solvent molecules (207 in the Mn^2+^-free enzyme). As far as the complete structure is concerned, the two Ape-MnP1 models are almost identical, as they superimpose with an overall rms deviation of 0.17 Å for the backbone atoms and of 0.42 Å for all but the five C-terminal residues (333–337).

The molecular structures of Ape-MnP1 and Cst-MnP1, which share 50% amino acid sequence identity, are characteristic of ligninolytic peroxidases (Fig. 1). They show minor differences that mainly affect the number and length of α-helices when compared with previously characterized short MnPs, such as *Pleurotus ostreatus* Pos-MnP4 (Fig. 1c). These differences are higher when compared to long MnPs, such as *Phanerochaete chrysosporium* Pch-MnP1 (Fig. 1d), due to the presence of an extra disulfide bridge and a longer C-terminal tail in the latter [24]. The overall fold of Ape-MnP1 and Cst-MnP1 is mainly helical (giving the enzymes a globular shape) with 13 and 15 α-helices, respectively, and one short antiparallel β-sheet at the N-terminus. Four disulfide bridges (Cys3–Cys15/Cys3–Cys15, Cys14–Cys280/Cys14–Cys279, Cys33–Cys115/Cys34–Cys115 and Cys244–Cys310/Cys243–Cys308 in Ape-MnP1/Cst-MnP1) present in all ligninolytic peroxidases (an extra disulfide bridge can be found in long MnPs as indicated above) contribute to maintain the protein conformation together with the two Ca^2+^ ions, also conserved in all ligninolytic peroxidases. These ions are coordinated in Ape-MnP1/Cst-MnP1 by Asp47/Asp48, Gly59/Gly61, Asp61/Asp63, Ser63/Ser65 and two water molecules in the case of Ca^2+^ positioned at the heme distal side; and by Ser172/Thr171, Asp189/Asp188, Thr191/Thr190, Ile194/Thr193 and Asp196/Asp195 in the case of Ca^2+^ located at the heme proximal side. The position of the above residues and other residues of interest described below are indicated on the amino acid sequences of Ape-MnP1, Cst-MnP1, Pch-MnP1 and Pos-MnP4 in Fig. S3 of Additional file 1. The Ca^2+^ environment is very well conserved in the MnP family. Minor differences can be observed at the surroundings of the distal Ca^2+^, where Ape-MnP1 and Cst-MnP1 showed the shortest loop (yellow) compared with short MnP4 (grey), long MnP1 (cyan) and extralong MnP6 (green) (Additional file 1: Fig. S4).

**Fig. 2:**
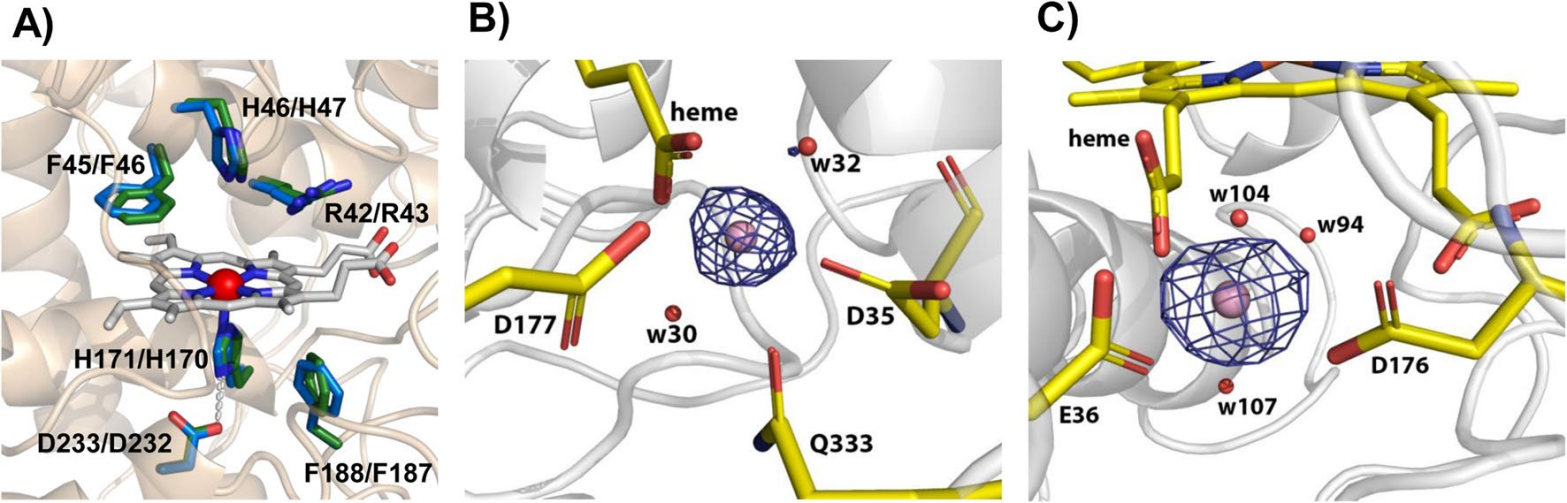
Heme region (**A**) and Mn^2+^-binding sites of Ape-MnP1 (**B**) and Cst-MnP1 (**C**). (**A**) Heme pocket of Ape-MnP1 (green) and Cst-MnP1 (blue), including His171/His170, Asp233/Asp232 and Phe188/Phe187 at the proximal side (below the heme plane); and His46/His47, Arg42/Arg43 and Phe45/Phe46 at the distal side (above the heme plane). Mn^2+^ binding in Ape-MnP1 (**B**) and Cst-MnP1 (**C**) confirmed by anomalous difference electron density maps contoured at 5*σ*, with the cation shown as a pink sphere

Regarding the heme pocket of both Ape-MnP1/Cst-MnP1 structures (Fig. 2a), it presents amino acids conserved in all ligninolytic peroxidases, including residues at the distal side (Phe45/Phe46, His46/His47 and Arg42/Arg43) involved in enzyme activation by H_2_O_2_ [34], and residues at the proximal side (Phe188/Phe187, Asp233/Asp232 and His171/His170), among which a histidine acts as the fifth ligand of the heme iron through its Nε2 (with a bond length of 2.16 Å/2.19 Å).

**Fig. 3:**
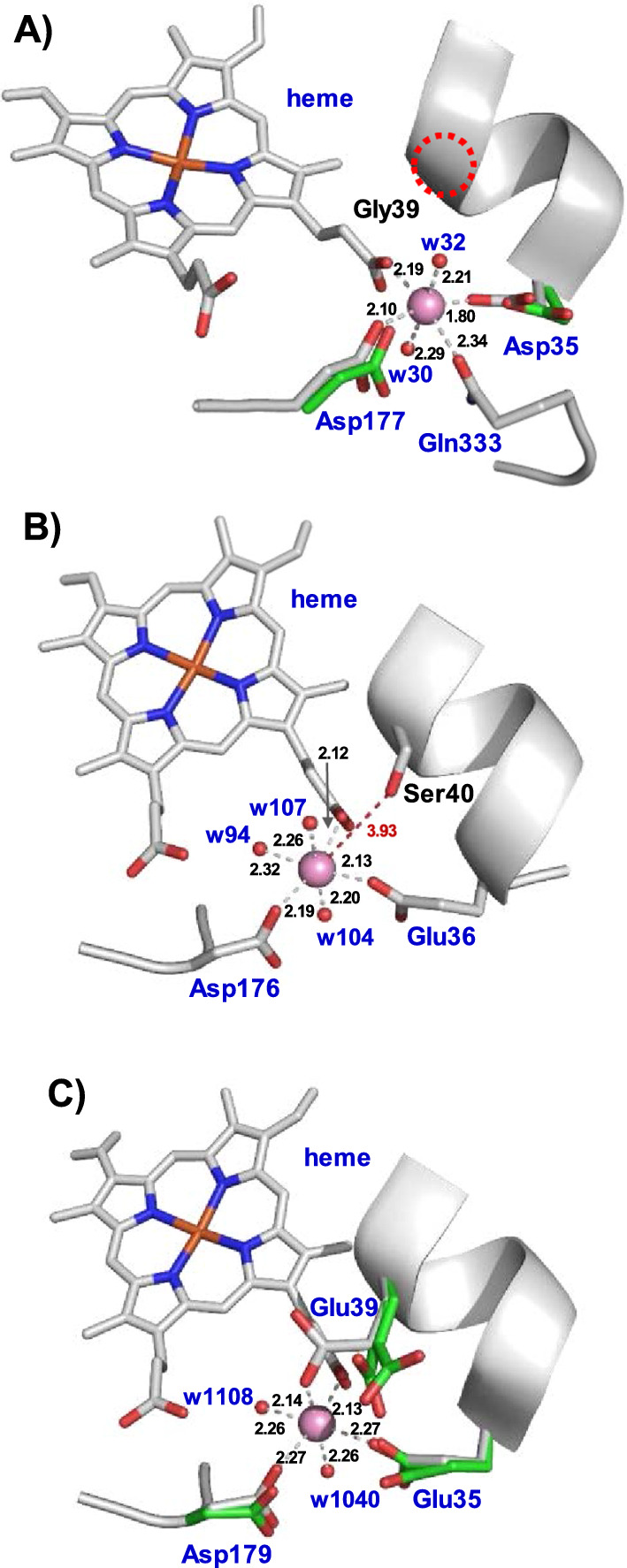
Mn^2+^-binding sites in the crystal structures of Ape-MnP1 (**A**), Cst-MnP1 (**B**) and Pch-MnP1 (**C**). Manganese coordination spheres (the cation shown as a pink sphere) include the internal heme propionate and: A) Asp35, Asp177, Gln333 and two water molecules (w30 and w32) in Ape-MnP1; B) Glu36, Asp176 and three water molecules (w94, w104 and w107) in Cst-MnP1; and C) Glu35, Glu39, Asp179 and two water molecules (w1040 and w1108) in the previously characterized Pch-MnP1. The green side chains superimposed in **A** and **C** correspond to the orientation of the amino acids in the structures obtained in the absence of Mn^2+^. Other residues previously predicted [28] to be coordinating manganese in Ape-MnP1 and Cst-MnP1 are indicated by black lettering

Mn^2+^ binding was confirmed by anomalous difference electron density maps (Fig. 2b, c). Surprisingly, the residues at the Mn^2+^ oxidation sites differ from previously characterized short and long MnPs, and also from those previously reported for these two novel MnPs based on in silico studies [28]. Figure 3 shows this site in detail, including bond distances among the Mn^2+^ ion and its ligands in Ape-MnP1 (A), Cst-MnP1 (B) and Pch-MnP1 (C). As observed in previously characterized MnPs, Mn^2+^ appears hexacoordinated by two water molecules and the carboxylates of two glutamates, one aspartate, and the internal heme propionate (Fig. 3c), a key contact for cation oxidation. Unlike known MnPs, the Ape-MnP1 model presents the manganese ion at the putative oxidation site coordinated by the carboxylates of two aspartates (Asp35 and Asp177), the internal heme propionate, two water molecules, and the side-chain carbonyl of Gln333 (positioned at the C-terminal end of the protein) instead of Gly39 as initially predicted [28] (Fig. 3a). Here, Asp35 and Gln333 play the role of Glu35 and Glu39, respectively, in the well-known Pch-MnP1 (Fig. 3c) and in previously characterized short and long MnPs. The different orientation of the Asp35 side chain in the structures of substrate-bound and substrate-free Ape-MnP1, and the absence of Gln333 in the crystal structure of the Mn-free enzyme, confirm the mobility of these residues, a behavior that has also been demonstrated for the two glutamates of the Mn^2+^ oxidation site in previously characterized MnPs [35]. Once coordinated, Gln333 also contributes to anchoring the C-terminal end of the enzyme, where this amino acid is located, restraining the mobility of this region of the protein.

Mn^2+^ oxidation site in Cst-MnP1 (Fig. 3b) also maintains the typical octahedral geometry of the other MnPs. However, this enzyme features only two amino acids (Glu36 and Asp176) as Mn^2+^ ligands together with three water molecules and the internal heme propionate. Ser40, which occupies the position of the second glutamate acting as a Mn^2+^ ligand in known long and short MnPs (Glu39 in Pch-MnP1, Fig. 3c), shows an increase in the Mn-residue bond length to 3.93 Å. This distance leaves Ser40 outside of the coordination sphere in Cst-MnP1, confirming that this residue is not involved in Mn^2+^ binding.

### pH, H_2_O_2_ and temperature stability of Ape-MnP1 and Cst-MnP1

**Fig. 4:**
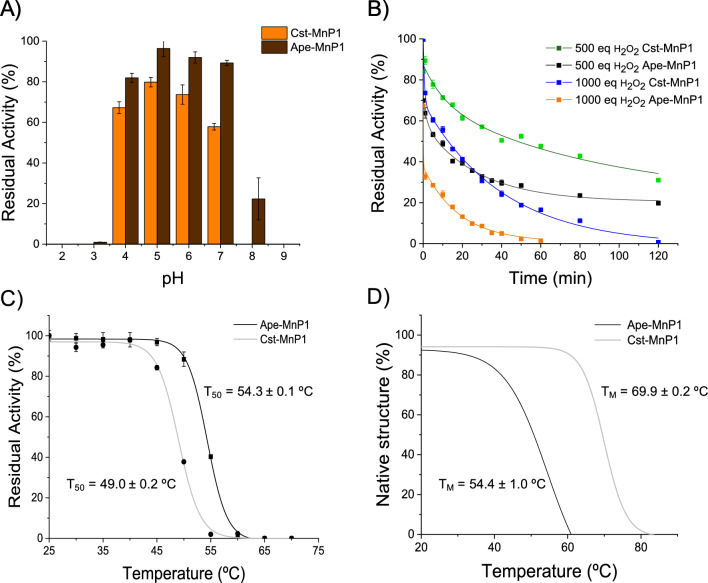
Effect of pH, H_2_O_2_ and temperature on Ape-MnP1 and Cst-MnP1 stability. **A** pH stability as residual activity after 24 h incubation in 100 mM Britton–Robinson buffer (pH 2–9) at 25 °C. **B** H_2_O_2_ stability assayed by incubating the enzymes with 500 and 1000 equivalents of H_2_O_2_ in 10 mM sodium acetate pH 5.5 at 25 °C, and further measurement of their residual activities. **C** Thermal effect on MnP activity after incubation in 10 mM sodium acetate, pH 5.5, for 10 min. **D** Effect of temperature on protein denaturation measured by CD at 222 nm. Residual activities in pH and H_2_O_2_ stability analyses were measured with 10 mM ABTS and 0.3 mM H_2_O_2_ in 100 mM sodium tartrate pH 3.0–3.5. Residual activities in thermal stability analyses were determined towards 10 mM MnSO_4_ in 100 mM sodium tartrate pH 4.0–5.0. *T*_m_ and *T*_50_ are provided. Means and standard deviations are shown

The stability of both peroxidases was analyzed against different factors. pH stability was evaluated by measuring residual activity between pH 2 and 9. As shown in Fig. 4a, both enzymes remained stable within the pH range of 4–7, retaining over 60% of the initial activity after 24 h. Outside this pH range, they were completely inactivated, with the exception of Ape-MnP1, which maintained 20% of its activity at pH 8.0.

Oxidative inactivation was examined by incubating the enzymes in the presence of increasing stoichiometric excess of H_2_O_2_. Curves of inactivation followed an exponential decay tendency (Fig. 4b) allowing the calculation of enzymes half-lives (*t*_1/2_) at each H_2_O_2_ concentration (Table 3). Ape-MnP1 was very sensitive to H_2_O_2_ excess, with half-lives of only 10 min and 45 s when incubated with 500 and 1000 eq of H_2_O_2_, respectively. By contrast, Cst-MnP1 was more stable, reaching half-lives of 40 and 21 min, respectively, and required longer H_2_O_2_ exposure times to completely lose its activity.

**Table 3:** Steady-state kinetic parameters for H_2_O_2_ reactions, and effect of H_2_O_2_ on MnPs stability

		Ape-MnP1	Cst-MnP1
*K*_M_^a^ (µM)		100 ± 14	40 ± 7
*k*_*c*at_ (s^−1^)		568 ± 55	265 ± 18
*k*_cat_/*K*_M_ (s^−1^·mM^−1^)		5650 ± 980	6620 ± 1270
*k*_i_ (µM)		(0.34 ± 0.06)·10^3^	(1.47 ± 0.29)·10^3^
*t*_1/2_ (min)^b^	500 eq	10	40
*t*_1/2_ (min)	1000 eq	0.75	21

^a^Reactions were conducted in 100 mM sodium tartrate, pH 5 for Ape-MnP1 and pH 4.5 for Cst-MnP1, at 25 °C, using MnSO_4_ as co-substrate. Data fitted to the equation describing inhibition

^b^Enzymes were incubated in sodium acetate, pH 5.5, at 25 °C with 500 and 1000 equivalents of H_2_O_2_. Residual activities were measured with ABTS in 100 mM sodium tartrate pH 3.0 and 0.3 mM H_2_O_2_

Thermal stability was analyzed by measuring both residual activities (using MnSO_4_ and 2,2′-azinobis[3-ethylbenzothiazoline-6-sulfonate], ABTS, as substrates) and circular dicroism (CD) melting profiles of the enzymes in the 25–90 °C range (Fig. 4c, d). Loss of secondary structure of Ape-MnP1 was consistent with the loss of its activity, with a melting temperature (*T*_m_) of 54.4 ± 1.0 °C and a *T*_50_ value of 54.3 ± 0.1 °C when measured against MnSO_4_. In the case of Cst-MnP1, the enzyme lost its activity before losing the protein structure probably due to local perturbations in the active site. Thus, a *T*_m_ of 69.9 ± 0.2 °C was observed while the *T*_50_ value against MnSO_4_ was of 49.0 ± 0.2 °C. *T*_50_ values of both enzymes toward ABTS (Additional file 1: Fig. S5) were similar to those observed against MnSO_4_, and activity was not recovered toward any of the substrates after incubating the enzymes again at room temperature (data not shown).

### Catalytic cycle and steady-state kinetic properties

The UV–visible spectral changes observed between the resting-state (RS) enzymes and their Compound I (CI) and Compound II (CII) intermediate states (Additional file 1: Fig. S6) confirmed that both MnPs follow the general catalytic cycle characterizing ligninolytic peroxidases [13]. Thus, CI formation was achieved after activation of the RS enzymes by H_2_O_2_, as revealed by a decrease in absorbance and a displacement of their maxima from 408 to 412 nm. In this state, the enzymes retain two oxidizing equivalents in the form of Fe^4+^ = O and porphyrin cation radical. CII was then obtained by one-electron enzyme reduction in the presence of 1 eq of ferrocyanide, resulting in spectra with maxima at 423 nm corresponding to the enzymes containing only one oxidation equivalent as a Fe^4+^ = O. Finally, the catalytic cycles of both peroxidases were completed after adding 10 eq MnSO_4_, recovering the absorbance properties of the RS enzymes, with a high-spin ferric heme ready to be activated again.

Substrate preference and catalytic properties of the enzymes were analyzed using Mn^2+^ and phenolic and non-phenolic aromatic compounds. The reactions were carried out at the optimum pH for each substrate (Additional file 1: Fig. S7) under saturating H_2_O_2_ concentrations using a molar excess of tenfold the Michaelis constant (*K*_M_) value (Table 3). Neither Ape-MnP1 nor Cst-MnP1 were active against the high redox potential non-phenolic lignin model compound 3,4-dimethoxybenzyl (veratryl) alcohol (VA) (1.45 V vs. NHE [37]). Instead, both peroxidases were able to oxidize Mn^2+^ as well as the low redox potential phenolic lignin model compound 2,6-dimethoxyphenol (DMP) (0.67 V vs. NHE [38]) and ABTS (0.69 V vs. NHE [39]) (Table 4). Interestingly, Ape-MnP1 showed ten- and threefold higher catalytic efficiency than Pos-MnP4 (representative of the short-MnP subfamily) at oxidizing DMP and Mn^2+^, respectively, and an efficiency toward this cation similar to that reported for Pch-MnP1 (representative of the long-MnP subfamily). In contrast, the efficiency of Ape-MnP1 oxidizing Mn^2+^ was twofold lower than that of the extra-long MnP of *Ceriporiopsis subvermispora* (Csu-MnP6). When the two novel MnPs were compared, Cst-MnP1 was found to be less efficient than Ape-MnP1 at oxidizing all the substrates assayed. This is especially significant for Mn^2+^ oxidation due to a lower substrate affinity, as revealed by a 23-fold higher *K*_M_ value.

**Table 4:** Steady-state kinetic constants for MnSO_4_, DMP and ABTS oxidation by native and some mutated MnPs^*a*^

		Ape-MnP1	Ape-MnP1 Q333P	Cst-MnP1	Cst-MnP1 S40A	Cst-MnP1 E36A	Pch-MnP1^*b*^	Pos-MnP4^*c*^	Csu-MnP6^*d*^
Mn^2+^	*K*_M_	58 ± 11	(1.0 ± 0.1)·10^4^	1310 ± 60	(3.2 ± 0.3)·10^4^	–	69	88 ± 4	9 ± 2
	*k*_cat_	256 ± 8	300 ± 9	244 ± 3	130 ± 6	–	300	125 ± 2	83 ± 5
	*k*_cat_/*K*_M_	4400 ± 860	29 ± 2	187 ± 10	4.2 ± 0.4	–	4325	1410 ± 60	9540 ± 650
DMP	*K*_M_	2880 ± 490	2900 ± 590	4650 ± 560	(1.3 ± 0.3)·10^4^	2120 ± 500	–	ns	–
	*k*_cat_	11.5 ± 0.8	12.2 ± 1.1	4.2 ± 0.2	7.31 ± 0.8	2.62 ± 0.17	–	ns	–
	*k*_cat_/*K*_M_	4.2 ± 0.5	4.2 ± 0.9	0.98 ± 0.12	0.57 ± 0.15	1.24 ± 0.30	–	0.4 ± 0	–
ABTS	*K*_M_	2730 ± 280	1890 ± 250	3250 ± 220	2990 ± 455	1900 ± 455	na	1560 ± 76	–
	*k*_cat_	146 ± 6	104 ± 4.6	60 ± 1.4	112 ± 4.6	67 ± 18	na	128 ± 3	–
	*k*_cat_/*K*_M_	53 ± 6	54.9 ± 7.6	18 ± 1.3	37.5 ± 5.9	35.5 ± 12.8	na	82 ± 3	–

^a^Kinetic parameters—*K*_M_ (µM), *k*_cat_ (s^−1^) and *k*_cat_/*K*_M_ (s^−1^·mM^−1^)—for MnSO_4_, DMP and ABTS reactions of Ape-MnP1 and its Q333P variant, Cst-MnP1 and its S40A and E36A variants, and Pch-MnP1, Pos-MnP4 and Csu-MnP6 as representative long, short and extra-long MnPs, respectively. Reactions were carried out in 100 mM sodium tartrate, at 25 °C, under H_2_O_2_ saturation (0.3 mM) at the optimal pH for each substrate. Data were fitted to the Michaelis–Menten equation. Means and 95% confidence limits of replicate assays. – no activity, na not available, ns not saturated

^b^From Mayfield et al. [40]

^c^From Fernández-Fueyo et al. [24]

^d^From Fernández-Fueyo et al. [41]

### Site-directed Ape-MnP1 and Cst-MnP1 variants at the Mn^2+^ oxidation site

Once the Mn^2+^ binding site was identified in the crystal structures of the novel MnPs (Figs. 2 and 3), and after confirming the ability of these enzymes to oxidize Mn^2+^ (Table 4), we analyzed the functionality of this site by mutating Gln333 in Ape-MnP1, and Glu36 and Ser40 in Cst-MnP1.

Substitution of Gln333 by proline in the Ape-MnP1-Q333P variant did not affect the activity of the enzyme towards DMP and ABTS (Table 4). However, this mutation led to a dramatic drop in the catalytic efficiency for Mn^2+^ oxidation (150-fold lower *k*_cat_/*K*_M_ compared with the wild-type enzyme) due to a decrease in substrate affinity (as a consequence of a significantly increase in the *K*_M_ value), whereas the enzyme turnover (catalytic constant, *k*_cat_) was hardly affected. This result revealed the relevance of Gln333 in the proper positioning of Mn^2+^ at the Ape-MnP1 manganese-oxidation site (Fig. 3a) during the catalysis.

As for Cst-MnP1, the E36A substitution in the Cst-MnP1-E36A variant resulted in the complete loss of the enzyme's ability to oxidize Mn^2+^, as expected given that Glu36 is one of the only two amino-acid residues (along with Asp176) that contribute to Mn^2+^ coordination in this enzyme (Fig. 3b). By contrast, this substitution had only a small effect on the enzymatic activity on DMP and ABTS, suggesting that phenols and other low redox potential compounds such as ABTS are oxidized at a catalytic site other than the heme internal propionate. Small changes on DMP and ABTS oxidation were identified in the Cst-MnP1-S40A variant. However, this substitution surprisingly led to a significant decrease in the affinity of the enzyme towards Mn^2+^ (24-fold higher *K*_M_) even though Ser40 is not involved in its binding. This effect was attributed to the destabilization produced by the loss of the H-bond found in the wild-type enzyme between the serine side chain and the heme internal propionate (Additional file 1: Fig. S8).

After experimentally analyzing the functionality of the catalytic site and determining the residues involved in Mn^2+^ binding and oxidation in Ape-MnP1 and Cst-MnP1, we renamed the new MnP subfamilies they belong to as MnP-DDQ (Asp/Asp/Gln Mn^2+^-oxidation site) and MnP-ED (Glu/Asp Mn^2+^-oxidation site), respectively, replacing the terms MnP-DGD (Asp/Gly/Asp) and MnP-ESD (Glu/Ser/Glu) previously proposed [28].

### MnP-DDQ and MnP-ED evolution

**Fig. 5:**
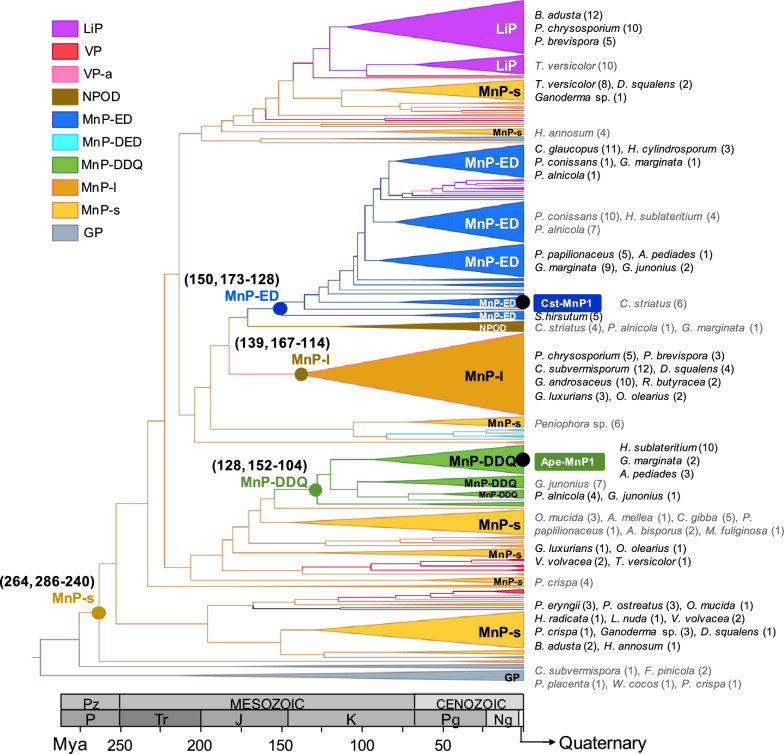
Dated phylogenetic tree of 336 POD sequences from 42 Agaricales, Polyporales, Amylocorticiales and Russulales species. Mean ages (and 95% highest posterior density, hpd, intervals) of the most recent common ancestors of four MnP subfamilies (in Ma) are indicated adjacent to the nodes. The color of the nodes refers to the MnP subfamily to which the reconstructed ancestor belongs, and the color of the branches indicates the type of the next ancestor (or current enzyme) in the tree: grey, generic peroxidase (GP); light orange, short MnP (MnP-s); dark orange, long MnP (MnP-l); red, versatile peroxidase (VP); pink, atypical VP (VPa); purple, lignin peroxidase (LiP); dark blue, MnP-ED; green, MnP-DDQ; light blue, MnP-DED; brown, NPOD. The position of Cst-MnP1 and Ape-MnP1 is shown inside boxes of the color of the subfamily they belong to. The name of the species and the number of PODs of each (sub)family (in parentheses) are shown at the tips of the branches

**Fig. 6:**
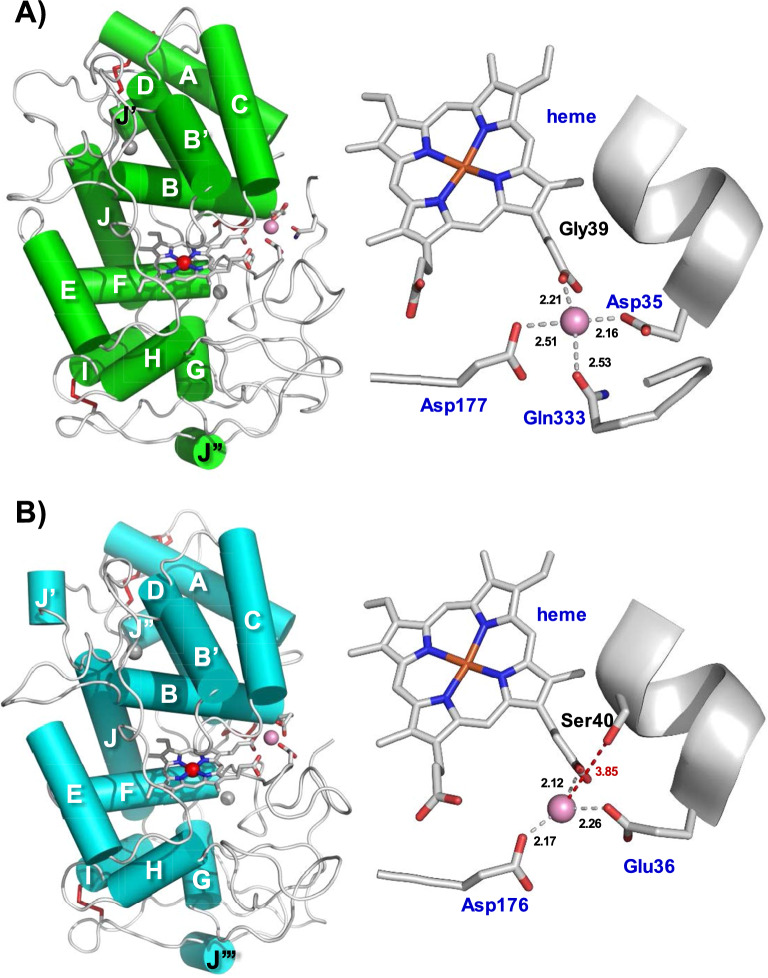
Homology models of the most recent common ancestors of the MnP-DDQ and MnP-ED subfamilies. Left, scheme of the overall structures of the ancestral MnP-DDQ (**A**) and MnP-ED (**B**) dated 128 Ma and 150 Ma, respectively (see Fig. 5), showing α-helices (cylinders), the heme cofactor (CPK sticks) with the iron center (red sphere), two structural calcium ions (grey spheres), disulphide bonds (red sticks), and one Mn^2+^ ion (pink sphere); Right, views of the Mn^2+^ coordination spheres with Asp35/Asp177/Gln333 and Glu36/Asp176 contributing to the Mn^2+^-binding site in the ancestral MnP-DDQ and MnP-ED, respectively (residues previously thought to be participating on Mn^2+^ binding are indicated in black font)

Given that the amino acids actually involved in Mn^2+^ oxidation in the MnP-DDQ and MnP-ED subfamilies (to which Ape-MnP1 and Cst-MnP1 belong, respectively) are not exactly the same as initially proposed, and that the latter were used as a reference to establish the evolutionary origin of these two MnP subfamilies [28], we decided to re-evaluate their origin based on the residues experimentally identified in the present study. For this purpose, we analysed the ancestral sequences reconstructed at the nodes of the phylogenetic tree in Fig. 5. The tree, consisting of 336 PODs identified in the genomes of 42 Agaricales, Polyporales, Amylocorticiales and Russulales species, is a partially collapsed version of that published by Ruiz-Dueñas et al., 2021 [28], and the ancestral POD sequences were those reported by these authors. In this tree, it is highlighted: (i) the origin of the short MnP subfamily, dated ~ 264 million years ago (Ma) (mean age, with 95% hpd-interval = 286–240 Ma) and long MnP subfamily, dated ~ 139 (167–114) Ma, both presenting a Mn^2+^ oxidation site formed by Glu/Glu/Asp residues; and (ii) the origin of the MnP-DDQ and MnP-ED subfamilies defined in the present study. This new analysis confirmed that the MnP-ED most recent common ancestor, which shares 77% amino acid sequence identity with Cst-MnP1, appeared in the Upper Jurassic ~ 150 (173–128) Ma; while the common ancestor of the MnP-DDQ subfamily, which shows an amino acid sequence identity of 70% with Ape-MnP1, appeared in the Lower Cretaceous ~ 128 (152–104) Ma, in both cases from short MnPs. The most efficient PODs appeared during the Cretaceous (Fig. 5), including not only MnP-DDQs but also long MnPs both able to oxidize lignin in manganese mediated reactions, as well as VPs and LiPs able to act directly on lignin through their solvent-exposed catalytic tryptophan [18, 42, 43]. Interestingly, these events took place during the diversification of angiosperms [44], characterized by a new type of lignin different from that of gymnosperms and more difficult to be degraded. The homology models obtained for the MnP-DDQ and MnP-ED most recent common ancestors, using the crystal structures of Ape-MnP1 and Cst-MnP1 as templates, show the heme internal propionate and Mn^2+^-binding residues at distances of 2.12–2.53 Å, compatible with their involvement in Mn^2+^ coordination (Fig. 6, right). This fact, together with the presence in these ancestors of the structural features characteristic of the most recent enzymes (helical fold, composition of the heme pocket, disulfide bridges, two structural calcium ions, etc.) (Fig. 6, left), suggests that the first MnP-DDQ and MnP-ED existing millions of years ago would have been able to oxidize Mn^2+^ with catalytic properties similar to those described for extant enzymes belonging to these MnP subfamilies.

### Lignin treatments with Ape-MnP1 and Cst-MnP1

**Fig. 7:**
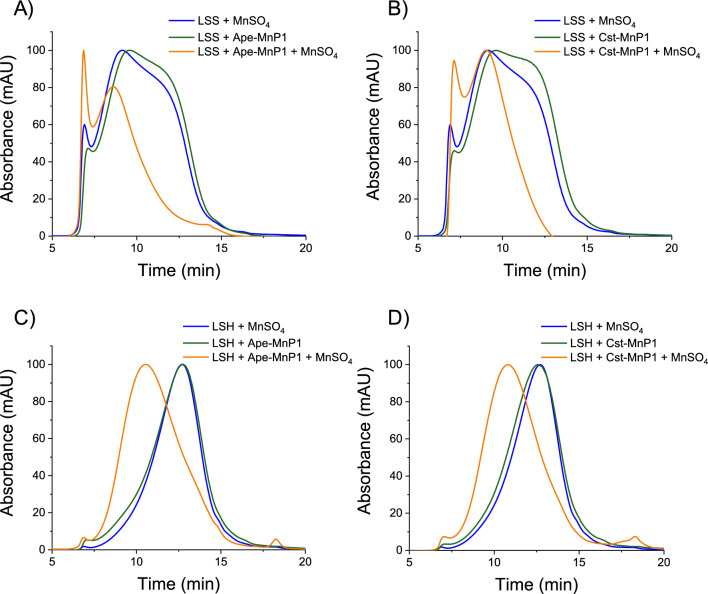
Molecular mass distribution of enzyme-treated and control softwood, LSS (**A**, **B**); and hardwood, LSH (**C**, **D**) lignosulfonates. Lignosulfonate treatments with 1 µM native enzymes (green lines) and lignosulfonate treatments with 1 µM native enzymes and 4 mM MnSO_4_ (orange lines), carried out by incubating 12 g·L-1 lignosulfonates in 50 mM sodium tartrate pH 5.0 for 24 h in the presence of 10 mM H_2_O_2_, were analyzed in a Superdex-75 column using 0.15 M NaOH/NaCl as mobile phase, and detection at 280 nm. Control lignosulfonates incubated with 4 mM MnSO_4_ are indicated with a blue line

To investigate the contribution of both MnPs to lignin transformation, hardwood and softwood lignosulfonates (water-soluble lignins) were treated with Ape-MnP1 and Cst-MnP1 for 24 h in steady-state experiments. Then, the reaction products were analyzed by size-exclusion chromatography (SEC) using a Superdex-75 column (with 0.15 M NaOH/NaCl as eluent, and 280-nm detection). The two enzymes were able to modify both lignosulfonates in the presence of manganese, which acts as a redox mediator, inducing changes in the distribution of their molecular masses (Fig. 7). Changes in the softwood lignosulfonate resulted in an increase in the higher molecular weight fraction eluting in the void volume, at 6.9 min, and in the disappearance of the large shoulder (Fig. 7a, b) of which most probably a part was incorporated into the high molecular weight fractions. For the hardwood lignosulfonate, the enzyme and manganese treatment resulted in a net increase of the average molecular mass (characterized by a shorter elution time) with both MnPs (Fig. 7c, d). Moreover, a small peak appeared at 18-min elution time, suggesting that these enzymes might contribute to formation of low molecular-weight structures. This might be considered as evidence of the ability of Ape-MnP1 and Cst-MnP1 to depolymerize lignin, since Fig. 7 mostly only shows repolymerization of both lignosulfonates after treatment with the two enzymes. On the other hand, minor changes in the chromatographic profiles were observed for the Ape-MnP1- and Cst-MnP1-treatments of both lignosulfonates in the absence of Mn^2+^. Even so, the differences were higher in the treatment of softwood lignosulfonate, which could be due to a possible direct attack of these enzymes to this more phenolic and less complex lignin compared to hardwood lignosulfonate.

## Discussion

### Production of novel manganese peroxidases under lignocellulolytic conditions

The adaptive response of plants to biotic and abiotic stresses has led to the evolution of the complex structure of the plant cell wall, primarily consisting of cellulose and hemicellulose polymers embedded in a lignin matrix that protect them from degradation [45]. Despite the recalcitrance of the lignin polymer, the structure of the plant cell wall is efficiently decomposed in nature by Agaricomycetes fungi [29]. For this purpose, they have developed complex enzymatic machineries responsible for a wide diversity of lifestyles related to lignocellulose degradation, such as white-rot and brown-rot decay of wood, forest-litter and grass-litter degradation, or decayed-wood degradation [28, 46, 47]. Among the enzymes found in these machineries, ligninolytic peroxidases of the LiP, MnP and VP families appear as a central component in lignin attack, playing a key role in wood degradation by white-rot fungi [12].

New peroxidase types rarely found in other Agaricomycetes have been recently identified in Agaricales (and some Russulales) [28]. This is the case of the until recently described as "atypical" MnPs, mostly found in leaf-litter and decayed-wood degrading fungi [28]. Despite their abundance in some Agaricales genomes, there are hardly any studies providing information about the production of these enzymes. Thus, so far, the expression of the enzymes herein renamed as MnP-EDs has only been reported in *Stereum hirsutum* growing on aspen wood [48], *Hebeloma cylindrosporum* and *Cortinarius glaucopus* transforming organic matter in forest soils [49], and *Agrocybe praecox* growing on cereal bran and forest litter [31]. As for MnP-DDQs, there are no studies reporting the presence of these enzymes in lignocellulosic cultures.

In the present study, we focused on the peroxidases of *A. pediades* and *C. striatus*, two Agaricales species that were selected as representatives of different lignocellulose-degrading lifestyles containing genes for novel MnPs in their genomes. Thus, *A. pediades* posseses genes encoding six MnP-EDs, three MnP-DDQs, in addition to one LiP; whereas the ligninolytic peroxidase set of *C. striatus* consists exclusively of MnP-EDs (9 genes) [28]. To mimic the natural (lignocellulolytic) growth conditions of these fungi, *A. pediades* was grown on wheat (*Triticum aestivum*) straw as it is a grassland-litter decomposer, whereas *C. striatus* was grown on beech (*Fagus sylvatica*) wood as it has been classified as a decayed-wood degrader [28]. Both species mobilized only part of their PODs. Thus one MnP-DDQ, one MnP-ED and one LiP isoenzymes were produced by *A. pediades*, whereas three MnP-ED isoenzymes were secreted by *C. striatus*. Furthermore, a differential expression of these enzymes was observed when compared to their production under non-lignocellulolytic conditions, this being a typical response of ligninolytic fungi to changes not only in nutritional but also in environmental conditions [50–55]. To better understand the role of these MnPs in lignin transformation, we selected the major PODs secreted by both fungi, MnP-DDQ JGI-ID#715400 (designated Ape-MnP1) and MnP-ED JGI-ID#1413095 (designated Cs-MnP1), for further structural–functional characterization.

### Structure–function relationship in the novel manganese peroxidases

Ape-MnP1 from *A. pediades* and Cst-MnP1 from *C. striatus* are structurally more closely related to the subfamily of short MnPs than to the subfamily of long MnPs. Thus, the former subfamily is characterized by presenting four structural disulfide bonds [22], while the latter contains five disulfide bonds and a longer C-terminal end that contribute to their catalytic and stability properties [24, 35]. From a functional point of view, the most significant structural differences of Ape-MnP1 and Cst-MnP1 are related to the residues forming the Mn^2+^ oxidation site. These residues are different from those previously identified in the homology models obtained for these enzymes [28] and from those (two glutamates and one aspartate) found in previously characterized short and long MnPs [22, 35]. As a consequence, using Ape-MnP1 and Cst-MnP1 as representatives of the subfamilies they belong to, we renamed both subfamilies as MnP-DDQ, with a manganese oxidation site made up of Asp/Asp/Gln; and MnP-ED, with a manganese oxidation site formed by Glu/Asp. It was surprising to find that the MnP-DDQ subfamily differs from previously characterized MnPs in two of the three residues involved in Mn^2+^ coordination, with Asp35 and Gln333 in Ape-MnP1 occupying the position of two glutamate residues in short and long/extra-long MnPs [22, 24, 35]. Moreover, this is the first MnP subfamily described that includes a residue of the C-terminal end contributing to the Mn^2+^ oxidation site. Comparison of the crystal structures of Mn-free Ape-MnP1 and Mn-bound Ape-MnP1 shows that the Asp35 side chain occupies different positions. In addition, Gln333 located at the C-terminal end does not even appear in the Mn-free Ape-MnP1 structure due to the predicted mobility of this region in the absence of the cation. The movements of these two residues witness the dynamic nature of the Mn^2+^ oxidation site in Ape-MnP1, a dynamic that is different from that reported for MnP of *P. chrysosporium* [35] and VP of *Pleurotus eryngii* [56], since the C-terminal tails of the latter are not involved in Mn^2+^ coordination.

Despite the structural differences observed in Ape-MnP1 and Cst-MnP1 compared with previously characterized MnPs, our analyses confirmed that both novel MnPs can oxidize Mn^2+^. It is of interest to note that this is the first experimental evidence of the oxidative capabilities of a MnP-DDQ. On the other hand, although a preliminary study had already shown the Mn^2+^ oxidizing ability of a MnP-ED produced by the litter decomposing Agarical *A. praecox* [31], an in-depth study on this MnP subfamily has not been performed until now.

Interestingly, Ape-MnP1 is among the most efficient MnPs oxidizing Mn^2+^. Thus, we found that its catalytic efficiency is higher (~ threefold) than those estimated for short MnPs [22], and similar to or slightly lower (up to ~ twofold) than those of long and extra-long MnPs [24, 40]. Conversely, Cst-MnP1 is among the least efficient MnPs oxidizing this cation (~ eightfold less efficient than short MnPs, and up to ~ 50-fold less efficient than long/extralong MnPs) [22, 24, 40]. Its *K*_m_ value (1310 µM) is similar to that of the *P. chrysosporium* MnP-E39A mutated variant (1900 µM) [25] which contains an oxidation site like that of Cst-MnP1 (consisting of a Glu and an Asp residues). Despite this low efficiency, it is interesting to note that, compared to other peroxidases, the Mn^2+^ oxidation activity of Cst-MnP1 is similar to that of two dye-decolorizing peroxidases (DyPs) from the ligninolytic fungi *Irpex lacteus* and *P. ostreatus* [57, 58], the enzyme of the latter species being suggested to participate in lignocellulose degradation by oxidizing lignin-derived compounds or modified lignin [51].

To further investigate the manganese oxidation sites of both enzymes, we produced the Ape-MnP1-Q333P, Cst-MnP1-E36A and Cst-MnP1-S40A variants. The kinetic constants of Ape-MnP1-Q333P confirmed that Gln333 participates in Mn^2+^ binding (a 172-fold decrease in Mn^2+^ affinity was observed for the mutated variant) but not in its oxidation (*k*_cat_ was hardly impaired). Accordingly, based on these results and the structural studies described above, we propose that the position of the Asp35 and Gln333 side chains in Mn-free Ape-MnP1 corresponds to an “open-gate” conformation of the active site, necessary for cation uptake. The subsequent reorientation of the side chains of these two residues pointing toward the Mn^2+^ ion in Mn-bound Ape-MnP1 corresponds to the “closed-gate” conformation, in which the enzyme oxidizes the cation in direct contact with the internal heme propionate after being activated by H_2_O_2_.

Regarding Cst-MnP1, the analysis of the E36A variant demonstrated that a single acidic residue (Asp176) at the Mn^2+^ oxidation site is insufficient for the catalytic process, as previously observed in a VP mutated variant containing only an Asp at this site [56]. This confirms that the catalytic site of Cst-MnP1, formed by Glu36 and Asp176, fufills the minimum requirements for Cst-MnP1 to act as a MnP. Moreover, this site is stabilized by Ser40, which contributes to anchor the internal heme propionate to the alpha-helix, where Glu36 is located, as inferred from the analysis of the S40A variant.

Besides activity, enzyme stability was also evaluated. pH stability of Ape-MnP1 and Cst-MnP1 was in the average of the short MnPs [22], being both enzymes less stable than the long/extra-long MnPs [24]. The fold of Cst-MnP1 was found to be more thermostable than Ape-MnP1 (*T*_m_ 69.9 °C vs. 54.4 °C, respectively). Although we have not been able to identify the structural determinants responsible for such stability, the one of Ape-MnP1 is similar to that of other extant PODs [22, 59], whereas the stability of Cst-MnP1 resembles that of ancestral PODs [60]. Nevertheless, higher fold thermostability of Cst-MnP1 did not correlate with the stability of its enzymatic activity, probably due to local perturbations in the active site responsible for the loss of activity at lower temperatures, compared to Ape-MnP1. We have also demonstrated that both Ape-MnP1 and Cst-MnP1 follow a time-dependent H_2_O_2_-mediated inactivation with decreasing half-life values at increasing concentrations of H_2_O_2_, as reported for other heme peroxidases [36, 61–63]. This is the result of a process that has been described as a suicide inactivation characterized by oxidation of amino-acid residues and heme destruction, leading to enzyme bleaching [64]. The differences in stability of the two enzymes are remarkable, Cst-MnP1 being much more stable than Ape-MnP1. However, an analysis of the structure of both peroxidases revealed no significant differences in the number and disposition of easily oxidizable residues (basically methionines) that could explain these differences. This means that other factors, such as a higher reactivity with H_2_O_2_ [36], could explain the low oxidative stability of Ape-MnP1 compared to Cst-MnP1.

### Enzymatic transformation of lignin

Having demonstrated the structural and functional properties of Ape-MnP1 and Cst-MnP1, it remained to determine whether these enzymes actually contribute to the transformation of the lignin polymer. The manganese-mediated ligninolytic activity of long MnPs was demonstrated years ago using gymnosperm (softwood) and angiosperm (hardwoods) synthetic lignins derived from the dehydrogenated polymerization of the corresponding monolignols as substrates [65] and suggested to proceed via phenoxy radicals of the phenolic lignin moieties. In these studies, in vitro depolymerization of phenolic lignin by MnP of *P. chrysosporium* occurred at short times (30 min incubation), after which repolymerization was predominant. Similarly, our treatments of hardwood and softwood lignosulfonates with Cst-MnP1 and Ape-MnP1 for 24 h in the presence of Mn^2+^ did not provide a clear evidence of their depolymerization, which occurs at shorter incubation times, but resulted in a net repolymerization as a consequence of radical coupling of the oxidation products [66]. We also detected some activity on lignosulfonates in the absence of Mn^2+^. This might be related to a possible ability of Cst-MnP1 and Ape-MnP1 to oxidize the terminal phenolic units of lignin in direct contact with the heme cofactor, as deduced from their ability to oxidize free phenols such as DMP. These peroxidases have two channels that give access to the heme active site where the phenolic units could be oxidized: (i) the main channel present in all heme peroxidases through which peroxide enters to activate the enzyme, that has been described as a low-efficiency site for phenols oxidization in VPs [67]; and (ii) the manganese channel that connects to the internal heme propionate, where not only the oxidation of Mn^2+^ but also of low redox potential substrates occurs, as reported for short MnPs [24].

Interestingly, the long-term repolymerization profiles of hardwood and softwood lignosulfonates by these novel MnPs in manganese-mediated reactions are similar to those observed for the *P. eryngii* VP and *P. chrysosporium* LiP-H8 acting via their solvent-exposed catalytic tryptophan [42, 43]. Similarly, the repolymerization profiles of both lignosulfonates due to the manganese-independent activity of MnP-DDQs and MnP-EDs on lignin phenolic units are comparable to those exhibited by VPs in catalytic tryptophan-independent reactions [42].

Consequently, our results suggest that, although some PODs are more efficient than others and their oxidation mechanisms are different [17] as a result of the evolution of their catalytic sites, all of them, including the novel MnP subfamilies herein described, contribute to lignin transformation with similar long-term effects. This in turn confirms the relevance of all POD families and subfamilies in the oxidation of different types of lignin.

## Conclusions

Basidiomycetes, as efficient lignocellulose degraders, play a pivotal role in environmental processes by acting on nutrient cycling and carbon sequestration rates. Understanding the composition and functionality of their oxidative enzymes provides a key to unraveling the mechanisms employed by these fungi in breaking down complex plant cell wall polymers, including lignin. In this work, we shed light on some of the intricate mechanisms underlying lignin transformation by Agaricales fungi through the characterization of two novel manganese peroxidase subfamilies present in their genomes. Through rigorous experimental analysis, we have identified the specific residues responsible for manganese oxidation in these enzyme subfamilies, demonstrated their lignin-modifying capabilities and elucidated their adaptive significance in the context of lignin degradation by revisiting their evolutionary origins. In this context, Ape-MnP1 was catalogued among the most efficient MnPs at oxidizing Mn^2+^. This study holds significance not only in advancing our understanding of Agaricales enzymatic systems, but also in showcasing the potential applications of these novel manganese peroxidases in the context of lignocellulose biorefineries, paving the way for the development of novel enzymatic tools for industrial lignin valorization.

## Methods

### *Agrocybe pediades* and *Cyathus striatus* secretomes

*A. pediades* AH40210 and *C. striatus* AH 40144 were obtained from the University of Alcalá Herbarium Culture Collection, Alcalá de Henares, Spain. Both fungi were grown in a medium containing (w/v) 2% malt extract, 2% glucose, 0.1% peptone and 2% agar, at 28 °C, and conserved at 4 °C.

The secretomes of *A. pediades* and *C. striatus* were collected from cultures in glucose–ammonium medium, wheat straw or beech wood as follows: both fungi were grown in 250 mL flasks containing 50 mL of glucose–ammonium medium [68] (liquid-state fermentation conditions); *A. pediades* was also grown in 250 mL flasks containing 4 g of chopped wheat (*T. aestivum*) straw (particle size ~ 5–20 mm long × 1–3 mm wide) soaked with 10 mL of distilled water; whereas *C. striatus* was grown in 250 mL flasks containing 9 g of chopped beech (*F. sylvatica*) wood (~ 20 mm long × 2–3 mm wide) soaked with 3 mL of distilled water (solid-state fermentation conditions). Inocula for the above culture media consisted of 4 mL of homogenized actively growing mycelium from glucose–ammonium cultures at 180 rpm and 28 °C (*A. pediades*) or 24 °C (*C. striatus*) washed and resuspended in sterile distilled water. Both liquid and solid-state fermentation cultures were grown at 28 °C (*A. pediades*) and 24 °C (*C. striatus*) under static conditions in the dark. Samples (entire flasks in triplicate) were collected after 14 days of incubation. Those from fungal cultures on lignocellulose were additionally treated with 80 mL distilled water at 180 rpm and 24 °C for 100 min. These samples and those from fungal cultures grown in glucose–ammonium medium were filtered under vacuum and the filtrates were used for proteomic analyses.

Total extracellular proteins in the above filtrates were treated and analysed as previously described for other fungal secretomes [69]. Briefly, samples were freeze-dried and resuspended in 200 mM sodium tartrate pH 5 and loaded in an SDS–PAGE gel. Protein bands were subjected to tryptic digestion and peptides were analysed in an LTQ-Orbitrap Velos mass spectrometer coupled to an Easy-nLC 1000 High-performance liquid chromatography (HPLC) system (Thermo Scientific).

Mass spectrometry (MS) data were then analysed with Proteome Discoverer (version1.4.1.14) (Thermo Scientific) using standardized workflows. Acquired spectra were searched against the catalogue of predicted proteins from the *A. pediades* AH40210 and *C. striatus*AH 40144 genomes, available at the JGI fungal genome portal MycoCosm (https://mycocosm.jgi.doe.gov/Agrped1 and https://mycocosm.jgi.doe.gov/Cyastr2, respectively), using SEQUEST search engine. Precursor and fragment mass tolerance were set to 10 ppm and 0.5 Da, respectively, allowing a maximum of two missed cleavages, carbamidomethylation of cysteines as a fixed modification, and methionine oxidation as a variable modification. Identified peptides were validated using Percolator algorithm [70] with a *q* value threshold of 0.01.

### Gene synthesis of wild-type peroxidases and their mutated variants

The DNA sequences encoding wild-type peroxidases JGI-ID#715400 from *A. pediades* and JGI-ID#1413095 from *C. striatus* (Ape-MnP1 and Cst-MnP1, respectively) were synthesized by ATG biosynthetics (Merzhausen, Germany) after codon optimization for *E. coli* expression using OPTIMIZER [71]. In addition, different mutations were designed in silico for these two peroxidases. The Q334P mutation was introduced into Ape-MnP1 by replacing the codon CAG with CCG. The changed codons in the mutated variants of Cst-MnP1 were GAA to GCG (E36A) and TCT to GCG (S40A). As with the wild-type enzymes, the mutated variants were also synthesized by ATG biosynthetics (Merzhausen, Germany). The synthesized DNA sequences were cloned into *Nde*I and *BamH*I restriction sites of the expression vector pET23b(+) (Novagen) and the resulting plasmids were transformed into *E. coli* DH5α for propagation. Plasmid purification was carried out using the High Pure Plasmid Isolation kit (Roche).

### Heterologous expression and in vitro activation

Native recombinant peroxidases and their site-directed variants were produced in *E. coli* BL21(DE3)pLysS after transformation with the corresponding plasmids. For that, cells were grown in Terrific Broth (TB) [72] containing 100 µg/mL ampicillin and 34 µg/mL chloramphenicol at 37 °C and 200 rpm until OD_600nm_ ~ 0.6. Expression was induced with 1 mM isopropyl β-D-1-thiogalactopyranoside for 4 h and then cells were harvested by centrifugation at 7000 rpm for 5 min at 4 °C. Bacterial pellets were resuspended in lysis buffer (50 mM Tris–HCl, pH 8.0, supplemented with 10 mM EDTA, 5 mM DTT and 2 mg/mL lysozyme) and treated with 0.1 mg/mL of DNaseI (Roche) at 4 °C for 45 min. After cell disruption by sonication, and subsequent centrifugation at 13,000 rpm for 30 min at 4 °C, the supernatant was discarded and the insoluble fraction, where the apoenzymes accumulated as inclusion bodies, was preserved. Protein aggregates were washed and solubilized in 8 M urea as described for other fungal oxidoreductases [73, 74].

Once solubilized, a factorial screening of conditions for in vitro folding of the recombinant native peroxidases was performed. Small-scale assays were carried out using 200 μL folding-mixture volumes in 96-well microplates, where the concentration of the components and folding conditions were modified. These included urea (0.16–1.2 M), GSSG (0–1.6 mM), hemin (5–15 μM), pH (7–9.5), temperature (4 or 25 °C), incubation time (24–72 h) and, in the case of Cst-MnP1, glycerol (0, 20 or 30%). All folding mixtures were prepared using constant protein (0.1 mg/mL), DTT (0.1 mM), EDTA (0.02 mM), buffer (50 mM Tris–HCl) and CaCl_2_ (5 mM) concentrations. Folding efficiency was checked by measuring activity toward 5 mM ABTS (resulting in ABTS^**·**+^ with *ε*_436_ 29,300 M^−1^·cm^−1^) in 100 mM sodium tartrate, pH 3.5, containing 0.1 mM H_2_O_2_. Optimal conditions for native Ape-MnP1 folding were 0.16 M urea, 0.8 mM GSSG and 15 μM hemin in 50 mM Tris–HCl, pH 9, and 24 h incubation at 4 °C, whereas those for native Cst-MnP1 were 0.6 M urea, 0.8 mM GSSG, 20% glycerol and 5 μM hemin in 50 mM Tris–HCl, pH 9.5, and 72 h incubation at 4 °C. The recombinant mutated variants were folded under the experimental conditions optimized for the native enzymes.

### Protein purification and quantification

Folding mixtures were concentrated (Pellicon and Amicon systems, with 10-kDa cutoff membranes, from Merck and Cole-Parmer, respectively) and ultracentrifuged (35,000 rpm, 4 °C, 1 h) for glycerol elimination, when present. Soluble fractions were dialyzed against 20 mM sodium acetate, pH 4.0, supplemented with 1 mM CaCl_2_, at 4 °C for 3 h, centrifuged at 8000 rpm for 15 min at 4 °C to remove misfolded precipitated protein, and re-dialyzed in 20 mM sodium acetate, pH 5.5, containing 1 mM CaCl_2_ at 4 °C. Then, the proteins were loaded into a 6-mL Resource-Q column (GE-Healthcare, USA) and eluted with a 0–400 mM NaCl gradient, at 2 mL/min flow, in 20 mM sodium acetate, pH 5.5, containing 1 mM of CaCl_2_. Finally, the purified enzymes were dialysed in the latter buffer containing 1 mM of CaCl_2_, and stored at − 80 °C. Protein purification was confirmed by SDS–PAGE in 12% polyacrylamide gels with 1% mercaptoethanol using Precision Plus Protein Dual Color Standards (Bio-Rad) and Coomassie R-250 staining. UV–visible spectra of the purified proteins were recorded in a Cary4000 spectrophotometer. Molar extinction coefficients of Ape-MnP1 (173,800 M^−1^·cm^−1^) and Cst-MnP1 (197,000 M^−1^·cm^−1^) were calculated according to Lambert Beer’s law, by measuring enzyme absorbance at 408 nm and referring to protein concentration, calculated as the average of the values obtained with NanoDrop (ThermoFisher NanoDrop 2000), Qubit (Invitrogen Qubit 3.0 Fluorimeter) and pyridine hemochrome assay [75].

### Crystallization, data collection and refinement

Crystallization of native peroxidases Ape-MnP1 and Cst-MnP1 was carried out with the sitting-drop vapor diffusion method at 295 K using a Cartesian Honeybee System (Genomic Solutions). Initial screening was conducted in 96-well sitting drop plates (Swissci, MRC) with the JBScreen Wizard (Jena Biosciences) crystallization kits. Each crystallization drop consisted of 0.2 µL of protein solution (10 mg/mL, in 20 mM sodium acetate, pH 5.5), containing 1 mM of CaCl_2_ and 0.2 µL of precipitant, equilibrated with 50 µL of the reservoir solution. Both peroxidases were co-crystallized in the presence of Mn^2+^ (added as MnSO_4_, 6 mM final concentration, to the crystallization solution) and Ape-MnP1 could also be crystallized in the absence of this cation. Thus, after individual optimization conditions, Cst-MnP1 crystals were obtained in 20% PEG 8000, 0.1 M Tris–HCl, pH 7, 0.3 M MgCl_2_, whereas Ape-MnP1 crystals were obtained in 30% PEG 2000 MME, 0.1 M sodium citrate, pH 4.2, in the presence and in the absence of Mn^2+^. For X-ray data collection, crystals were transferred into a crysolution containing the mother liquor supplemented with 25% (v/v) PEG 400, and frozen in liquid nitrogen prior to data acquisition.

Complete data sets were collected at the BL13-XALOC beamline of the ALBA Synchrotron (Cerdanyola del Vallès, Spain). To validate the presence of the manganese ion, additional data sets were collected at a wavelength of 1.74138 Å. Data were processed using XDS [76] and merged and scaled with AIMLESS [77], from the CCP4 package [78]. The structures were solved by molecular replacement using the crystal structure of the *Trametes cervina* lignin peroxidase (PDB entry 3Q3U) [79] as the search model and the program MOLREP implemented in the CCP4 package [78]. The initial models were refined using REFMAC [80] and alternating manual rebuilding with COOT [81]. The structures were analyzed and validated using MolProbity [82]. Figures illustrating protein structures were drawn with PyMOL (Schrödinger).

### pH, H_2_O_2_ and temperature stability

pH stability was determined by measuring the residual activity of the purified enzymes (1 µM) after 24 h incubation in 100 mM Britton–Robinson buffer, within pH range 2 to 12, at 25 °C.

H_2_O_2_ stability was assayed by measuring the enzymatic activity after incubation of 1 µM enzyme in 10 mM sodium acetate, pH 5.5, at 25 °C with 500 and 1000 equivalents of H_2_O_2_. Residual activities were measured every 5 min until 1 h of incubation. The experimental data of residual activity versus time at each H_2_O_2_:MnP ratio were fitted to an exponential decay model as follows:

$$\begin{aligned} & {\text{MnPactivity}}\,\left( \% \right) = {\text{MnPact}}_0 \cdot {\text{e}}^{ - \lambda t} \\ & t_{1/2} = \frac{\ln (2)}{\lambda } \\ \end{aligned}$$

where *λ* is de activity decay constant and *t*_1/2_ the enzyme half-life.

Temperature stability was analyzed by monitoring both the residual activity and secondary structure loss after protein incubation at different temperatures. Activity decrease was analyzed after the incubation of 1 µM enzyme in 10 mM sodium acetate, pH 5.5, in the range of 25–70 °C for 10 min. *T*_50_ values were calculated as the temperature at which the enzymes lost 50% of their activity after 10 min incubation. Protein denaturation was studied by CD from 25 °C to 90 °C at 30 °C/h using a J-720 spectropolarimeter (Jasco, Oklahoma City, OK, USA) equipped with a temperature controller and a thermostated cell holder. Samples containing 6 µM enzyme in 20 mM sodium acetate, pH 5.5, and 1 mM CaCl_2_ were measured using a cell with 0.1 mm optical path length. The thermal melting profile was presented and the *T*_m_ value was calculated as the temperature at the midpoint of the unfolding transition.

Residual activities in pH, H_2_O_2_ and thermal stability analyses were measured using 10 mM ABTS as a substrate, 10 nM enzyme and 0.3 mM H_2_O_2_ in 100 mM sodium tartrate, pH 3.0–3.5. Activity towards 10 mM MnSO_4_ at pH 4.0–5.0 was also measured during thermal stability analyses. For each enzyme, the highest activity after 1 min incubation (at any pH or temperature) was taken as 100%, and the residual activities under the different conditions were provided as percentages of this maximal value.

### MnPs catalytic cycle

Formation of Compound I (CI), Compound II (CII) and resting-state (RS) intermediates of the native enzymes was followed by analyzing spectral changes with an Agilent 8453 UV–visible diode-array spectrophotometer in a 200–800 nm wavelength range. Spectra were recorded in 100 mM sodium tartrate pH 5.0 at 25 °C. For CI formation, the native enzymes (~ 0.5 µM) were mixed with 2 equivalents of H_2_O_2_ until spectra maxima were stabilized at 412 nm (Soret maximum of CI). CII formation was ensured by mixing CI intermediates with 1 equivalent of potassium hexacyanoferrate (II) (ferrocyanide) and followed at 423 nm (Soret maximum of CII). Finally, CII reduction to RS was measured at 408 nm (Soret maximum of RS) upon mixing with 10 equivalents of MnSO_4_.

### Steady-state kinetic constants

Kinetic characterization was conducted using MnSO_4_, ABTS, DMP and VA. MnSO_4_ oxidation was followed by monitoring the formation of a Mn^3+^–tartrate complex (*ε*_238_ 6500 M^−1^ cm^−1^). ABTS oxidation was followed by the formation of the ABTS cation radical (ABTS^·+^ *ε*_436_ 29,300 M^−1^ cm^−1^). DMP oxidation led to its dimerization forming the colored product coerulignone (*ε*_469_ 55,000 M^−1^ cm^−1^). VA oxidation was monitored by the formation of veratraldehyde (*ε*_310_ 9300 M^−1^ cm^−1^).

Enzymatic activities were measured as initial velocities using a Thermo Scientific Biomate5 spectrophotometer, at 25 °C, optimum pH and saturating H_2_O_2_ concentration. The optimum pH for oxidation of the above substrates was determined at saturating concentrations of ABTS and DMP (10 mM each) in 100 mM Britton–Robinson buffer (pH 2–10), and MnSO_4_ (10 mM) in 100 mM sodium tartrate over the pH range 2.5–5.5, using 10–40 nM enzyme and 0.1 mM H_2_O_2_. The assays were conducted in 1 mL reaction volume.

To determine H_2_O_2_ optimal concentrations, kinetic constants were analyzed in 1 mL of reaction mixture using 10 mM MnSO_4_ in 100 mM sodium tartrate pH 4.5–5.0. Kinetic parameters were obtained by fitting the data to the Michaelis–Menten equation *v*/[*E*] = (*k*_cat_ · [*S*])/(*K*_m_ + [*S*]), or the equation describing inhibition *v*/[E] = (*k*_cat_ · [*S*])/(*K*_m_ + [*S*] + (1 + ([*S*]/*K*_*i*_))).

### Steady-state treatment of lignin

To evaluate MnPs participation in lignin transformation, 12 g L^−1^ hardwood (*Eucalyptus grandis*) and softwood (*Picea abies*) lignosulfonates provided by G.E. Fredheim (Borregaard AS, Sapsborg, Norway) were treated in 50 mM tartrate pH 5, at 25 °C for 24 h with and without the addition of 4 mM MnSO_4_. Final concentration of enzyme was 1 µM, added in two equal doses at 0 and 12 h. H_2_O_2_ was added continuously with a syringe pump until a final concentration of 10 mM. Control treatments were performed under the same conditions but in the absence of enzyme. Changes in the molecular-mass distribution of treated lignosulfonates were analyzed by SEC at 280 nm using a Superdex-75 column (HR-10/30, 3000–70000/100000 Da range; GE Healthcare) with 0.15 M NaOH/NaCl as the mobile phase and a flow rate of 0.5 mL·min^−1^. Blue dextran (Serva, Heindelberg, Germany) was used to determine the exclusion volume of the column and a kit of sulfonated polystyrenes sodium salt standards with main peaks (Mp) in the 891–976,000 Da range (PSS, Mainz, Germany) was used for calibration and mass determination.

### Re-evaluation of the evolutionary origin of the MnP subfamilies

The reconstruction of ancestral ligninolytic peroxidases recently reported by our research group [28] was used to re-evaluate the evolutionary origin of the different MnP subfamilies. The most probable amino-acid sequences at the nodes of the ligninolytic peroxidases phylogeny were analyzed regarding the presence of relevant residues identified in Ape-MnP1 and Cst-MnP1 sequences, including the amino acids forming the Mn^2+^-oxidation site and others such as the proximal histidine involved in heme iron coordination and distal residues responsible for enzyme activation by H_2_O_2_. Then, theoretical molecular models of selected ancestral manganese peroxidases were generated by using the programs implemented by the automated protein homology-modeling server SWISS-MODEL [83]. For that, the crystal structures of Ape-MnP1 and Cst-MnP1 obtained in this work were used as templates. The analysis of these models, mainly focused in the location of the amino acids forming the Mn^2+^ binding site, enabled us to identify the more recent common ancestors of the subfamilies the MnPs under study belong to.

### Supplementary Information

Supplementary Material 1.

## Data Availability

All the data supporting the conclusions of this article are included within the article and its supplementary information. Additional information can be provided by the corresponding authors under request.

## Abbreviations

ABTS: 2,2′-Azinobis(3-ethylbenzothiazoline-6-sulfonate)
CI: Compound I
CII: Compound II
CD: Circular dicroism
DMP: 2,6-Dimethoxyphenol
DTT: Dithiothreitol
EDTA: Ethylenediaminetetraacetic acid
GSSG: Oxidized glutathione
HPLC: High-performance liquid chromatography
*k*_cat_: Catalytic constant
*k*_cat_/*K*_m_: Catalytic efficiency
*K*_m_: Michaelis constant
LiP: Lignin peroxidase
LSH: Hardwood lignosulfonate
LSS: Softwood lignosulfonate
MnP: Manganese peroxidase
POD: Class II peroxidase
RS: Resting state
VA: 3,4-Dimethoxybenzyl (veratryl) alcohol
VP: Versatile peroxidase
