# Supplementary material for: Structure–function characterization of two enzymes from novel subfamilies of manganese peroxidases secreted by the lignocellulose-degrading Agaricales fungi Agrocybe pediades and Cyathus striatus
Source: Biotechnol Biofuels Bioprod. 2024 Jun 1;17:74. doi: 10.1186/s13068-024-02517-1 (PMC11144326; doi:10.1186/s13068-024-02517-1)
Supplement: Supplementary file 1 — Supplementary Material 1. [file 13068_2024_2517_MOESM1_ESM.doc]

**SUPPLEMENTARY INFORMATION**

**Structure-function characterization of two enzymes from novel subfamilies of manganese peroxidases secreted by the lignocellulose-degrading Agaricales fungi *Agrocybe pediades* and *Cyathus striatus***

María Isabel Sánchez-Ruiz, Elena Santillana, Dolores Linde, Antonio Romero, Angel T. Martínez and Francisco Javier Ruiz-Dueñas*

Centro de Investigaciones Biológicas Margarita Salas (CIB), CSIC, Ramiro de Maeztu 9, E-28040 Madrid, Spain

*Corresponding author: [fjruiz@cib.csic.es](mailto:fjruiz@cib.csic.es)

**Supplementary Figures**

This Supplementary Information includes: **Figure S1**. *In vitro* activation and purification of Ape-MnP1 and Cst-MnP1; **Figure S2**. Electronic absorption spectra of Ape-MnP1 (A) and Cst-MnP1 (B); **Figure S3**. Multiple alignment of the amino acid sequences of Ape-MnP1, Cst-MnP1, Pos-MnP4 and Pch-MnP1; **Figure S4**. Environment of the exposed loop of the distal Ca^2+^ in MnPs; **Figure S5**. Temperature stability of the recombinant enzymes measured with ABTS; **Figure S6**. Electronic absorption spectra of the intermediate states of the catalytic cycle of Ape-MnP1 (A) and Cst-MnP1 (B). **Figure S7**. Optimal pH for oxidation of MnSO_4_, ABTS and DMP by Ape-MnP1 (A) and Cst-MnP1 (B); and **Figure S8**. LIGPLOT diagram of heme interactions in Cst-MnP1 (PDB 8qwt).

**Figure S1.** ***In vitro* activation and purification of Ape-MnP1 and Cst-MnP1.** Screening of the optimal urea (0.16-2.80 M) and GSSG (0-1.6 mM) concentrations for the *in vitro* folding of Ape-MnP1 (**A**) and Cst-MnP1 (**B**) as part of a multifactorial design in which 0.1 mg/mL protein was incubated at 4ºC in the presence of 0.1 mg/mL DTT, 0.02 mM EDTA and 5 mM CaCl_2_, with hemin concentration, pH and glycerol indicated for each enzyme (the color goes from light blue, for *in vitro* activation conditions leading to the highest percentage of enzyme activity, to dark blue, for conditions where no enzyme activity is detected). Resource-Q chromatograms of Ape-MnP1 (**C**) and Cst-MnP1 (**D**) recombinant enzymes showing the elution profiles at 280 nm (gray line) and 410 nm (black line) and NaCl gradient (dashed line). SDS–PAGE of molecular-mass markers (of 250, 150, 100, 75, 50, 37, 25 and 20 kDa, from top to bottom) and purified enzymes are indicated in the inset.

**Figure S2.** **Electronic absorption spectra of Ape-MnP1 (A) and Cst-MnP1 (B).** UV-visible spectra of the enzymes at the resting state, with indication of the Soret band (408 nm) and CT1 (637 nm) and CT2 (502 nm) charge-transfer bands (inset).

**Figure S3.** **Multiple alignment of the amino acid sequences of Ape-MnP1, Cst-MnP1, Pos-MnP4 and Pch-MnP1.** Conserved catalytic and other relevant residues are indicated with different colors including: eight cysteines (ten in Pch-MnP1) (cyan) forming four disulfide bridges (five in Pch-MnP1); nine ligands (green) of two structural Ca^2+^ ions; two active site histidines (dark gray); residues (orange) forming the Mn^2+^ oxidation site; and several active site conserved residues (light gray). Alignment was prepared using Clustal Omega (EMBL’s European Bioinformatics Institute). Amino acid numbering starts at the first residue of the mature protein. Symbols below indicate full conservation of the same (*) or equivalent residues (:) and partial residue conservation (.).

**Figure S4.** **Environment of the exposed loop of the distal Ca^2+^ in MnPs**. Ape-MnP1 and Cst-MnP1 are indicated in yellow, *P. ostreatus* MnP4 in grey, *P. chrysosporium* MnP1 in cyan and *C. subvermispora* MnP6 in green.

**Figure S5. Temperature stability of Ape-MnP1 and Cst-MnP1 measured using ABTS as substrate.** Samples were incubated for 10 min at different temperatures in 10 mM sodium acetate pH 5.5. Residual activities were measured against 10 mM ABTS in 100 mM sodium tartrate, pH 3.0-3.5 using 0.3 mM H_2_O_2_. T_50_ values are provided. Means and standard deviations are shown.

**Figure S6. Electronic absorption spectra of the intermediate states of the catalytic cycle of Ape-MnP1 (A) and Cst-MnP1 (B).** Maximum absorbance of resting state (RS- black), compound I (CI – dark gray) and compound II (CII – light gray) are indicated.

**Figure S7. Optimal pH for the oxidation of MnSO_4_, ABTS and DMP by Ape-MnP1 (A) and Cst-MnP1 (B).** Reactions were initiated by H_2_O_2_ addition (0.3 mM) and activities were measured at pH 2-9 in 100 mM Britton-Robinson buffer (100 mM sodium tartrate buffer for MnSO_4_ oxidation). Residual activities (means and standard deviations) were expressed as percentages of the maximal activity for each substrate.

**
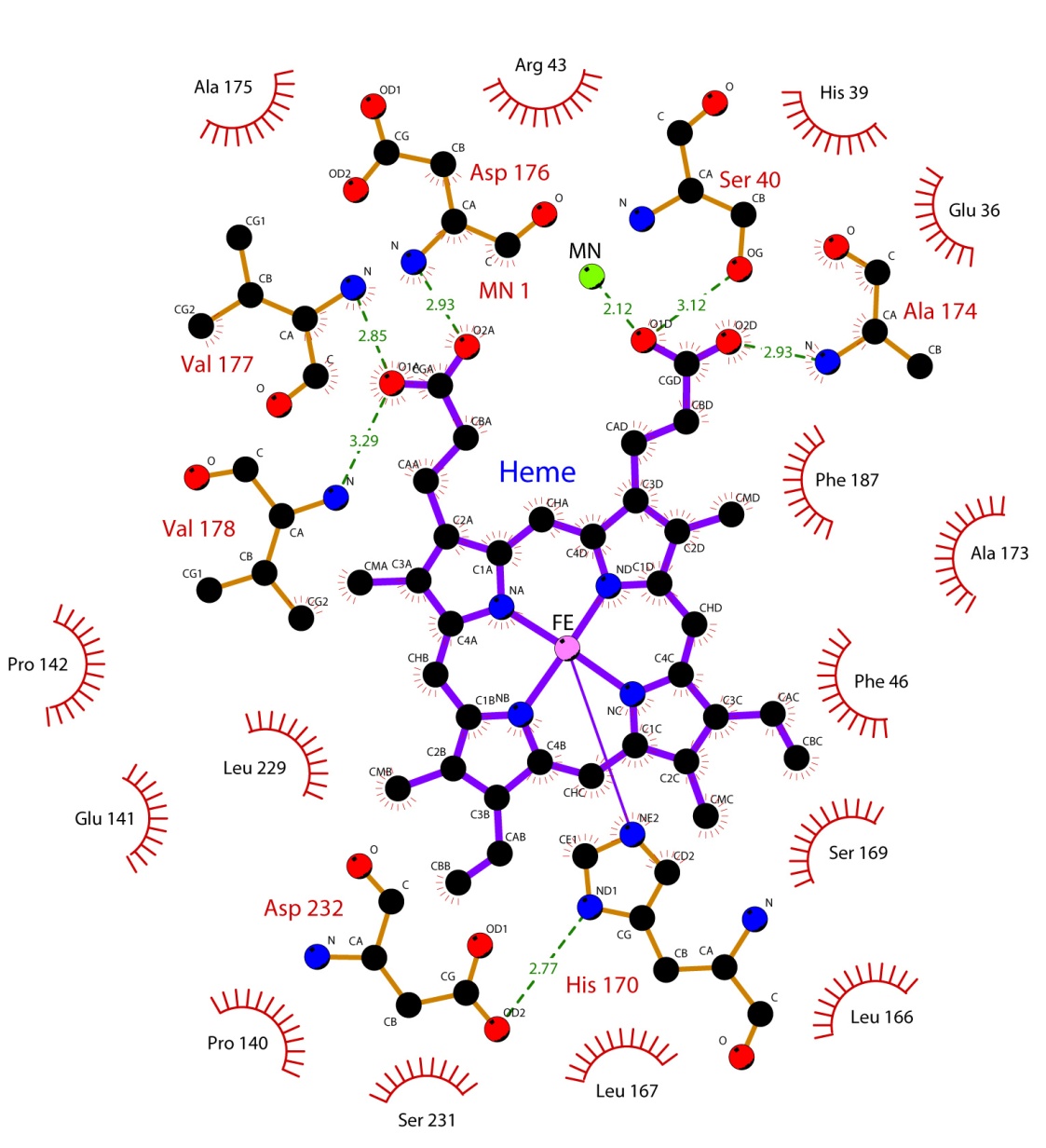
**

**Figure S8. LIGPLOT diagram of heme interactions in Cst-MnP1 (PDB 8qwt).** HBPLUS was used to calculate hydrogen bonds and hydrophobic contacts (the latter are interpreted by following the spokes protruding from a ligand atom toward a protein residue, which is shown as an arc). Heme bonds are depicted in purple line and those of the protein in brown line; and hydrogen bonds, and their lengths in Å, are shown in green. The heme iron and manganese ion are represented as pink and light green spheres, respectively.
